# 

**Published:** 1843-01-21

**Authors:** 


CONTENTS.

Lectures on the Treatment of Wounds. Delivered at the Hôtel-Dieu, of Paris. By M. Roux. Lecture 1. . . . . . . . 1

Letter from Dr. Meigs, . . . . . . . 3

Dr. Wm. Pepper's Case of Aneurism of the Thoracic Aorta, opening into the left Bronchus; with remarks, . . . . . . . 4

*University of Pennsylvania.* Clinic of Professor Horner.

Luxation of Cervical Vertebræ, . . 5

Dr. W. Poyntell Johnston's Clinic.

Case 1. Capsulo-lenticular cataract of the left eye—aborescent—very slightly tremulous, the result of a blow, . . . . . . 5

Case 2. Purulent ophthalmia, succeeding labour—reduction of inflammaiion—chronic conjunctivitis for five years—reappearance in its purulent form—destruction of right eye—vascular keratitis of left eye, 6

Case 3. Variolous ophthalmia of right eye at 7 weeks of age.—Obliteration of pupil.—Formation of artificial pupil.—Partial Vision.—Complete amaurosis of left eye, from wound three years since.—Exophthalmia following section of internal and external recti muscles, . . . . . . 7

*Jefferson Medical College.*

Service of Dr. Mütter. . . . . . . 8

*Philadelphia Hospital.* Professor Pancoast's Clinic.

Case 1. Dislocation of the head of the radius, . . . . . . . . . . . . . . 9

Case 2. Injury of the Head, . . . . . 9

Case 3. Strangulated Crural Hernia, . . 10

*Bibibliographical Notice.*

Clinical Surgery of the Hospital of La Pitié. By J. Lisfranc. Vol. I. Paris, 1842. 11

*Editorial Remarks,* . . . . . . . . . 12

Muriate of Ammonia in Hemicrania, . . 12

Liquor Taraxaci, . . . . . . . . . 12

